# MicroRNA-107 enhances radiosensitivity by suppressing granulin in PC-3 prostate cancer cells

**DOI:** 10.1038/s41598-020-71128-1

**Published:** 2020-09-03

**Authors:** Hua-Cheng Lo, Jia-Hao Hsu, Liang-Chuan Lai, Mong-Hsun Tsai, Eric Y. Chuang

**Affiliations:** 1 Division of Urological Surgery, Department of Surgery, Tri-Service General Hospital Songshan Branch, National Defense Medical Center, Taipei, 10581 Taiwan, ROC; 2 Graduate Institute of Biomedical Electronics and Bioinformatics, Department of Electrical Engineering, National Taiwan University, Taipei, 10617 Taiwan, ROC; 3 Graduate Institute of Physiology, College of Medicine, National Taiwan University, Taipei, 100 Taiwan, ROC; 4 Bioinformatics and Biostatistics Core, Center of Genomic and Precision Medicine, National Taiwan University, Taipei, 10055 Taiwan, ROC; 5 Institute of Biotechnology, National Taiwan University, Taipei, 10617 Taiwan, ROC; 6 Biomedical Technology and Device Research Laboratories, Industrial Technology Research Institute, Hsinchu, 31040 Taiwan, ROC

**Keywords:** Radiotherapy, Prostate

## Abstract

Prostate cancer is the second leading cause of cancer-related death worldwide. Radiotherapy is often applied for the treatment, but radioresistance is a challenge in some patients. MicroRNAs have been reported to be involved in the DNA damage response induced by ionizing radiation and recent studies have reported microRNA-mediated radiosensitivity. In the present study, we found microRNA-107 (miR-107) enhanced radiosensitivity by regulating granulin (GRN) in prostate cancer (PC-3) cells. MiR-107 was downregulated and GRN was upregulated in response to ionizing radiation in PC-3 cells. Overexpression of miR-107 and knockdown of *GRN* promoted the sensitivity of PC3 cells to ionizing radiation. By rescue experiments of GRN, we revealed that radiosensitivity enhanced by miR-107 can be attenuated by GRN overexpression in PC-3 cells. Furthermore, we showed miR-107 enhanced radiation-induced G1/S phase arrest and G2/M phase transit, and identify delayed apoptosis by suppressing p21 and phosphorylation of CHK2. Collectively, these results highlight an unrecognized mechanism of miR-107-mediated GRN regulation in response to ionizing radiation and may advance therapeutic strategies for the treatment of prostate cancer.

## Introduction

Prostate cancer (PCa) is the second most common male cancer worldwide, with an estimated 1.4 million cases and 381,000 deaths in 2016^1^. In the USA, an estimated 164,690 new cases and 29,430 deaths occurred from PCa in 2017^2^. Radiotherapy is currently one of the common definitive treatment choices for patients with localized or locally advanced PCa, and also plays a role in palliative treatment for patients with bony metastases. However, successful radiotherapy mainly relies on tumor radiosensitivity and the tolerance of normal tissues. Radiotherapy in PCa is not innocuous and is usually accompanied by urinary and bowel side effects. Furthermore, despite technological advances in radiation therapy delivery, the rate of biochemical/clinical relapse among PCa patients who have undergone radiotherapy remains unfortunately high^3^ and is the leading cause of treatment failure. Therefore, exploration and clarification of potential targets or molecular mechanisms in tumor radioresistance can help to improve the effectiveness of radiotherapy and lower incidence of side effects in the future.

MicroRNAs (miRNAs) are a class of small, non-coding RNA molecules that play significant roles in cellular regulatory mechanisms, such as cell differentiation, survival and proliferation by binding to the 3′-untranslated region (3′-UTR) of a target gene’s mRNA, leading to translational inhibition or degradation^4^. MiRNAs modulate diverse cellular and developmental processes including adhesion, migration, invasion proliferation, inflammation, apoptosis, and cell cycle, most of which constitute crucial hallmarks of cancer^5^. Recently, the role of miRNAs in regulating tumor radiosensitivity has been exhaustively investigated, and considerable evidence has shown a close link between miRNA expression and radioresistance in cancers^6–10^. However, functional mechanisms of miRNAs in radioresistance are still largely unknown.

MicroRNA-107 (miR-107) is a member of the “miR-15/107 gene superfamily” which includes miR-15a/b, miR-16, miR-103, miR-107, miR-195, miR-424, miR-497, miR-503, and miR-646^11^. The expression of miRNAs from this group is moderate to high in a wide variety of human tissues, including cells derived from the endoderm, mesoderm, and ectoderm^11,12^. The miR-15/107 superfamily regulates several fundamental processes including inflammation, metabolism, stress response, angiogenesis, and cell cycle regulation. Furthermore, dysregulation of the miR-15/107 family has been implicated in pathological processes and associated with neoplasia, neurodegenerative diseases and heart disease^11,13^. Studies have shown miR-107 is differentially expressed in various cancer cells, such as being upregulated in gastric^14^ and liver cancer^15^, and downregulated in breast^16^, renal^17^, cervical^18^, glioma^19^ and non-small cell lung cancers^20^. Thus, miR-107 can act as either a tumor suppressor or an oncomir. In addition, it has been shown that miR-107 is downregulated in response to ionizing radiation (IR)^21–23^. However, whether miR-107 plays a role in the response to IR in PCa cells is unclear.

Granulin (GRN) is a pleiotropic growth factor with multiple given names, including acrogranin, granulin-epithelin precursor, gp88, progranulin, proepithelin, and PC cell–derived growth factor^24^. GRN is a potent growth factor and mitogen involved in many human cancers^24,25^, including PCa. Pan and colleagues showed that the expression of GRN was much fewer with a smaller fraction of cells in normal prostate tissue than in prostatic intraepithelial neoplasia (PIN) and invasive adenocarcinoma^26^. Monami and colleagues demonstrated GRN plays a critical role in PCa by promoting cell motility of hormone-refractory PCa and contributes as an autocrine growth factor to the transforming phenotype by regulating invasion and anchorage-independent growth^27^. Although previous studies have shown that miR-107 can suppress GRN expression posttranscriptionally in some cancer cells, including neuroglioma^28^ and PCa cells^29^, the regulation of GRN expression in response to IR is still unclear.

Based on literature surveys, and the results of a miRNA microarray study performed by our laboratory^30^, we suggest miR-107 may play a critical role in radiosensitivity in PCa. In this study, we reveal miR-107 is downregulated and GRN expression is increased in PC-3 cells after exposure to IR. MiR-107 overexpression in tumor cells efficiently suppressed GRN expression and proliferative activity. We further demonstrate that either overexpression of miR-107 or *GRN* knockdown by shRNA prominently inhibited the survival of PC-3 cells after IR exposure in clonogenic assays. Overexpression of GRN attenuated miR-107-induced growth inhibition and cell survival after IR, demonstrating that GRN is a key effector in miR-107 modulated radiosensitivity. Furthermore, repression of GRN by either miR-107 or by shRNA suppressed p-CHK2 and p21 activity, leading to G1/S arrest, G2/M transit, and delayed apoptosis. Our study provides new findings of connections between miR-107 and GRN in modulating radiation-induced cell cycle arrest and apoptosis, and enrich the known relationship between miRNAs and radiosensitivity.

## Results

### Altered expression of miR-107 in response to radiation in PC-3 cells

**Figure 1:**
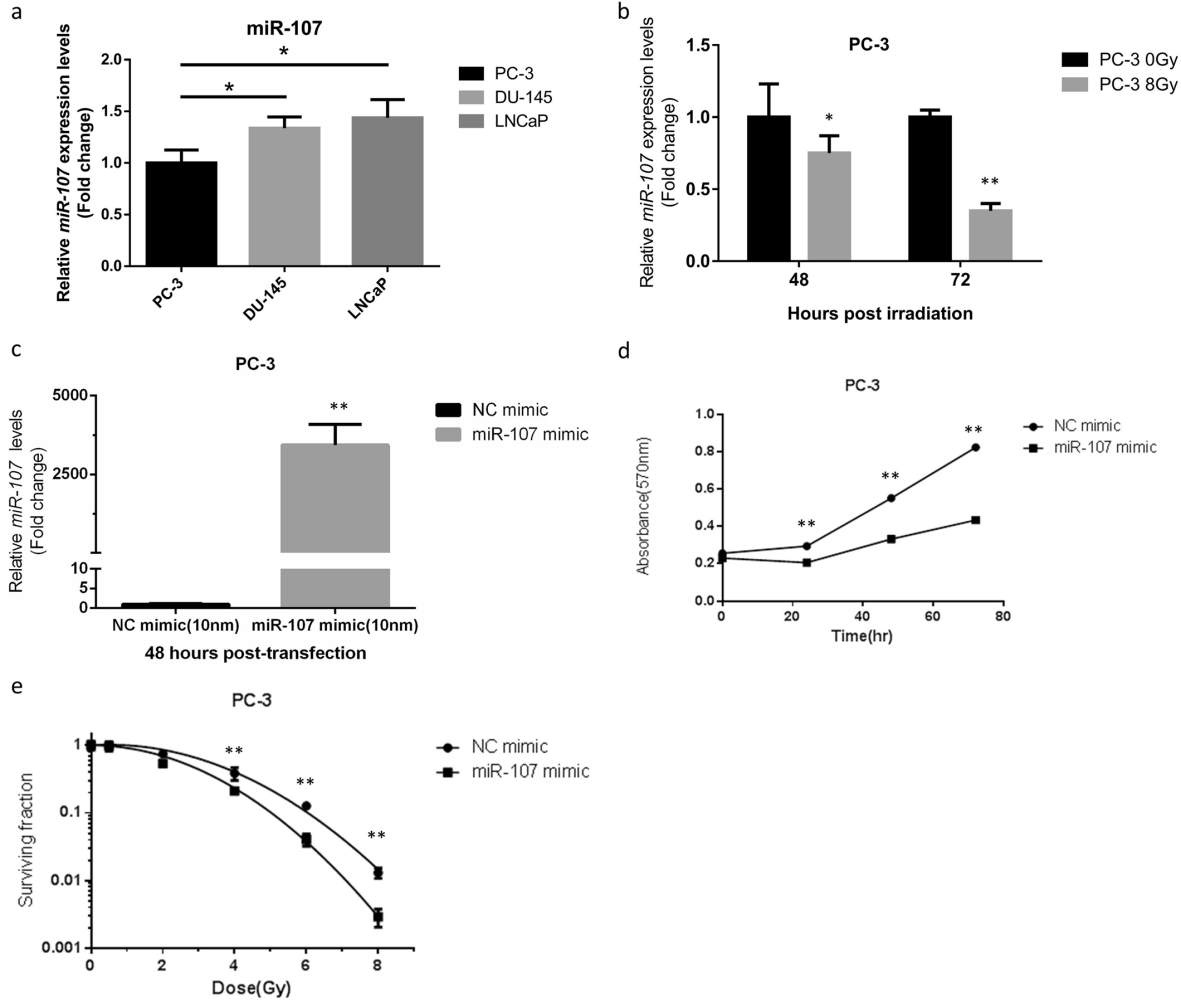
Expression levels of miR-107 were down-regulated in PC-3 cells in response to IR and overexpression of miR-107 enhanced radiosensitivity of PC-3 cells. (**a**) Relative expression levels of miR-107 in PCa cells. (**b**) Relative expression levels of miR-107 in PC-3 cells at the indicated time points after exposure to 8 Gy, detected by qRT-PCR. (**c**) MiR-107 expression after transfection of PC-3 cells with miR-107 mimic or negative control (NC). (**d**) Cell proliferation and (**e**) colony formation of PC-3 cells transfected with miR-107 or NC after IR. Data were representative of more than three independent experiments, with each performed in triplicate. (*p < 0.05; **p < 0.01).

To assess expression profiles of miR-107 in prostate cancer cell lines, a quantitative real time polymerase chain reaction (qRT-PCR) analysis was used for comparison of endogenous expression patterns, which showed a relative low level of miR-107 expression in PC-3 cells (Fig. 1a). Some studies had shown miR-107 expression was down-regulated in response to ionizing radiation (IR) in several cancers, including PCa cells^23^, and thus we chose PC-3, an androgen-independent PCa cell line obtained from patients with bony metastatic lesions, to investigate its reaction after IR. The levels of miR-107 expression profile were determined at 48 and 72 h post-IR (8 Gy) using qRT-PCR (Fig. 1b). MiR-107 expression was significantly downregulated in response to IR compared to sham irradiation, which implied miR-107 may play a role in radiosensitivity.

### Overexpression of miR-107 inhibited proliferation and enhanced radiosensitivity of PC-3 cells

Since miR-107 was found to be downregulated significantly in response to IR in PC-3 cells, we tested the hypothesis that overexpression of miR-107 could alter cancer cell behavior in response to IR. Using transient transfection with a miR-107 mimic, we overexpressed miR-107 in PC-3 cells. MiR-107 levels were significantly increased in the miR-107 mimic group compared to negative control (NC) miRNA (Fig. 1c). Overexpression of miR-107 significantly suppressed proliferation in the miR-107 mimic-transfected PC-3 cells compared to NC groups in MTT assays (Fig. 1d). To further confirm the role of miR-107 in radiosensitivity, clonogenic assays were performed. Results of the clonogenic assays also showed the surviving fractions of PC-3 cells after IR were lower in cells transfected with miR-107 mimic versus NC miRNA (Fig. 1e, Supplementary Fig. 1). Moreover, we found the radiosensitivity enhanced by miR-107 mimic is also significant in DU-145 cells (Supplementary Fig. 2a). These results were consistent with the hypothesis that miR-107 overexpression decreases cell proliferation and suppresses cell survival/viability after IR, resulting in increased radiosensitivity of PCa cells, including PC-3 and DU-145 cells.

**Figure 2:**
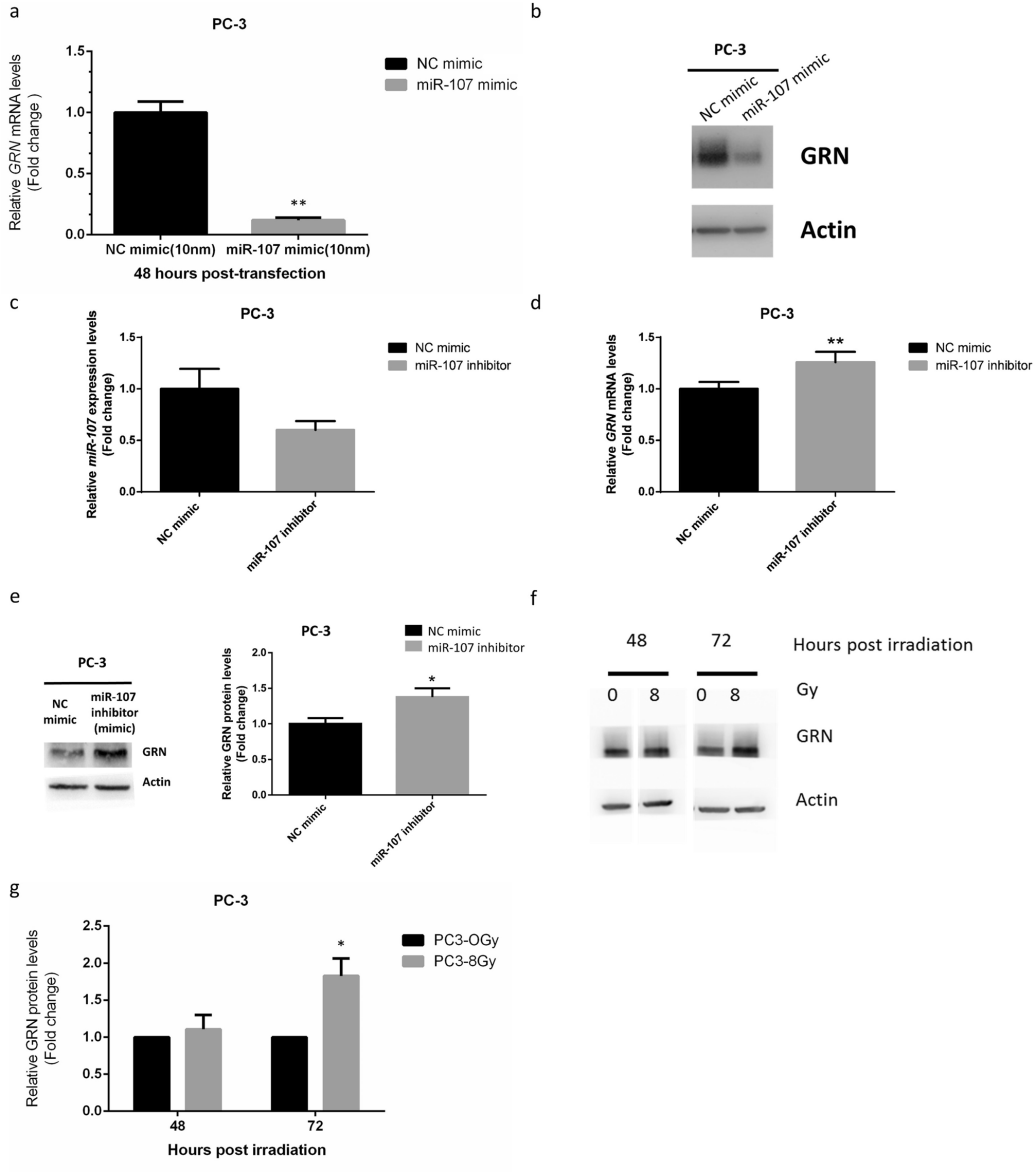
MiR-107 overexpression down-regulated GRN, whereas miR-107 inhibition and IR up-regulated GRN. Relative expression of GRN (**a**) mRNA and (**b**) protein after miR-107 treatment for 48 h. (**c**) MiR-107 expression after transfection of PC-3 cells with miR-107 inhibitor or negative control (NC). Relative expression of GRN (**d**) mRNA and (**e**) protein after miR-107 inhibitor treatment for 48 h. (**f**–**g**) GRN protein expression levels at 48 and 72 h after exposure to 8 Gy irradiation. Data were representative of more than three independent experiments, with each performed in triplicate. (*p < 0.05; **p < 0.01).

### Granulin is a direct target of miR-107 and is suppressed by miR-107 in PC-3 cells

The cellular functions of miRNAs are revealed through their target genes. To understand how miR-107 modulates radiosensitivity, the target genes of miR-107 were predicted using online web tools, including miRSystem^31^, TargetScan, miRBase, miRDB and miRTarBase. miRSystem and miRTarBase showed the prediction that GRN is a target for miR-107. Wang and colleagues used dual luciferase reporter assays to document that miR-107 can regulate GRN, whose sequences complementary to the 5′ seed region of miR-107 are present in the open reading frame (ORF) conserved from zebrafish to humans^28^. At the same time, we were interested in investigating GRN because of its close relationship with tumorigenesis and malignancy in PCa^26,27^. Hence, we investigated and established that GRN is regulated by miR-107 in PC-3 cells, and that expression of GRN is also relatively higher in PC-3 cells compared with LNCaP and DU-145 cells (Supplementary Fig. 3). After miR-107 mimic treatment, the mRNA and protein levels of GRN were significantly suppressed in PC-3 (Figs. 2a and 2b). In addition, miR-107 mimic treatment can also downregulate mRNA level of GRN in DU-145 cells (Supplemental Fig. 2b). Moreover, after the miR-107 inhibitor treatment (Fig. 2c), mRNA and protein levels of GRN were significantly increased (Fig. 2d,e). These results collectively demonstrated that GRN is a direct target of miR-107, and is downregulated by miR-107 in PCa cells, at least in PC-3 and DU-145 cells.

### GRN expression increases in response to irradiation in PC-3 cells

To further identify the role GRN in radiobiological effects, we exposed PC-3 cells to IR. The expression levels of GRN in PC-3 cells following IR were verified by western blot analysis. The GRN protein expression was significantly upregulated in response to IR in PC-3 cells in a time-dependent manner (Fig. 2f,g). These results indicated that GRN is a potential biomarker involved in response to IR in PCa cells, and that it might be associated with radiosensitivity.

### Knockdown of GRN sensitizes PC-3 cells to IR

**Figure 3:**
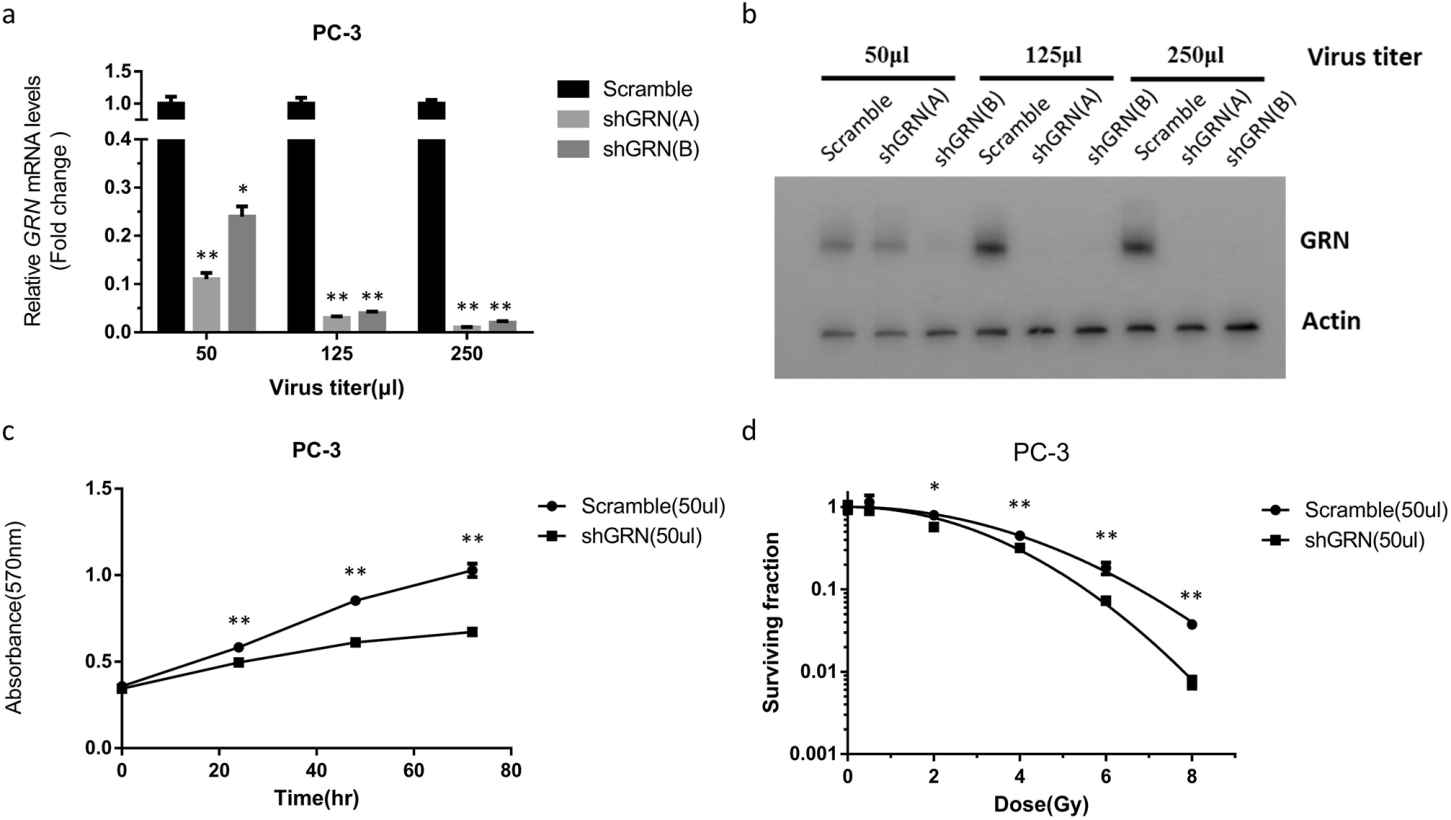
Knockdown of *GRN* enhanced radiosensitivity of PC-3 cells. (**a**) GRN expression was repressed by shRNAs at the mRNA level. qRT-PCR was conducted to quantify GRN expression after transfection with shRNAs into PC-3 cells. (**b**) GRN expression was repressed by shRNAs at the protein level. Western blotting was performed after transfection of PC3 cells with shRNAs. (**c**) Cell proliferation and (**d**) colony formation of PC3 cells transfected with shGRN or scramble shRNA after exposure to 8 Gy IR. Data were representative of more than three independent experiments, with each performed in triplicate. (*p < 0.05; **p < 0.01).

As GRN has been shown to be a target of miR-107, and overexpression of miR-107 enhanced the sensitivity of PC-3 cells to IR, we subsequently attempted to determine whether miR-107 sensitizes PC-3 cells to IR by directly targeting GRN. To validate this, *GRN* was knocked down by several specific short hairpin RNAs (shRNAs) in PC-3 cells, and cellular proliferation and colony formation ability after IR were evaluated. As shown in Fig. 3a,b, both mRNA expression and protein level of GRN were significantly suppressed by shGRN(A) compared to the scramble shRNA. Thus, shGRN(A) was selected to knock down *GRN* expression in PC-3 cells and was hereafter referred to as “shGRN”. After transfection with shGRN, PC-3 cells had significantly lower cellular proliferation than after transfection with the scramble shRNA (Fig. 3c). After IR, the surviving fractions of PC-3 cells transfected with shGRN were markedly lower than those transfected with scramble shRNA cells in clonogenic assays (Fig. 3d, supplementary Fig. 4). These data revealed knockdown of *GRN* increased the radiosensitivity of PC-3 cells. Taken together, the above results confirmed miR-107 enhanced the radiosensitivity of PC-3 cells by targeting the expression of GRN.

### GRN overexpression rescues proliferation and attenuates radiosensitivity induced by miR-107 in PC-3 cells

**Figure 4:**
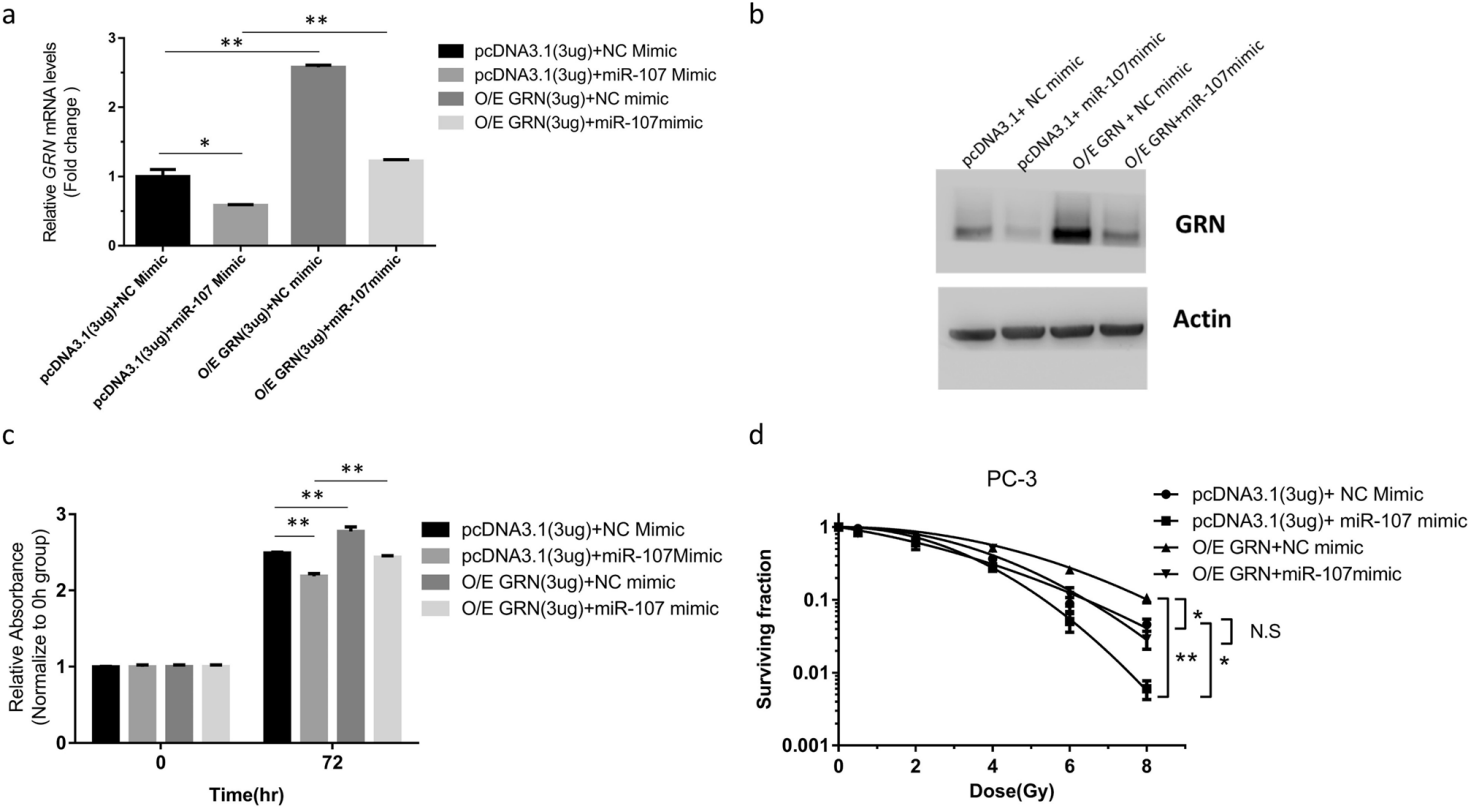
GRN overexpression (O/E) rescued proliferation and neutralized radiosensitivity enhanced by miR-107 mimic in PC-3 cells. Relative expression of GRN mRNA (**a**) and protein (**b**) after cotransfection with NC mimic + pcDNA3.1, miR-107 mimic + pcDNA3.1, NC mimic + pcDNA3.1-GRN, or miR-107 mimic + pcDNA3.1-GRN, respectively. (**c**) Cell proliferation and (**d**) colony formation of PC-3 cells cotransfected with the above RNAs and plasmids after IR. Data were representative of more than three independent experiments, with each performed in triplicate. (*p < 0.05; **p < 0.01). pcDNA3.1 represents the empty vector pcDNA™3.1^(−)^. pcDNA3.1-GRN represents the vector pcDNA™3.1^(−)^ containing the *GRN* gene construct. NS represents the none significant.

To clarify whether anti-proliferation and radiosensitivity regulated by miR-107 is mediated through suppressing GRN, a pcDNA3.1-GRN expression plasmid was constructed to conduct a rescue experiment. The pcDNA3.1-GRN vector and pcDNA3.1 control vector were transfected into PC-3 cells with the NC mimic or miR-107 mimic, and the GRN mRNA and protein expression levels were examined. After transfection with pcDNA3.1-GRN expression plasmid, the relative expression levels of *GRN* mRNA (Fig. 4a) and GRN protein (Fig. 4b) were significantly increased. The cellular proliferation suppressed by miR-107 mimic was regained in cells overexpressing GRN (O/E GRN) as compared to control cells transfected with miR-107 mimic (Fig. 4c). After IR, the surviving fraction was increased in cells O/E GRN as compared to control cells transfected with miR-107 mimic (Fig. 4d, Supplementary Fig. 5). This rescue experiment revealed GRN overexpression can attenuate the growth inhibition and radiosensitivity induced by miR-107.

### MiR-107 alters cell cycle distribution and enhances delayed apoptosis in PC-3 cells

**Figure 5:**
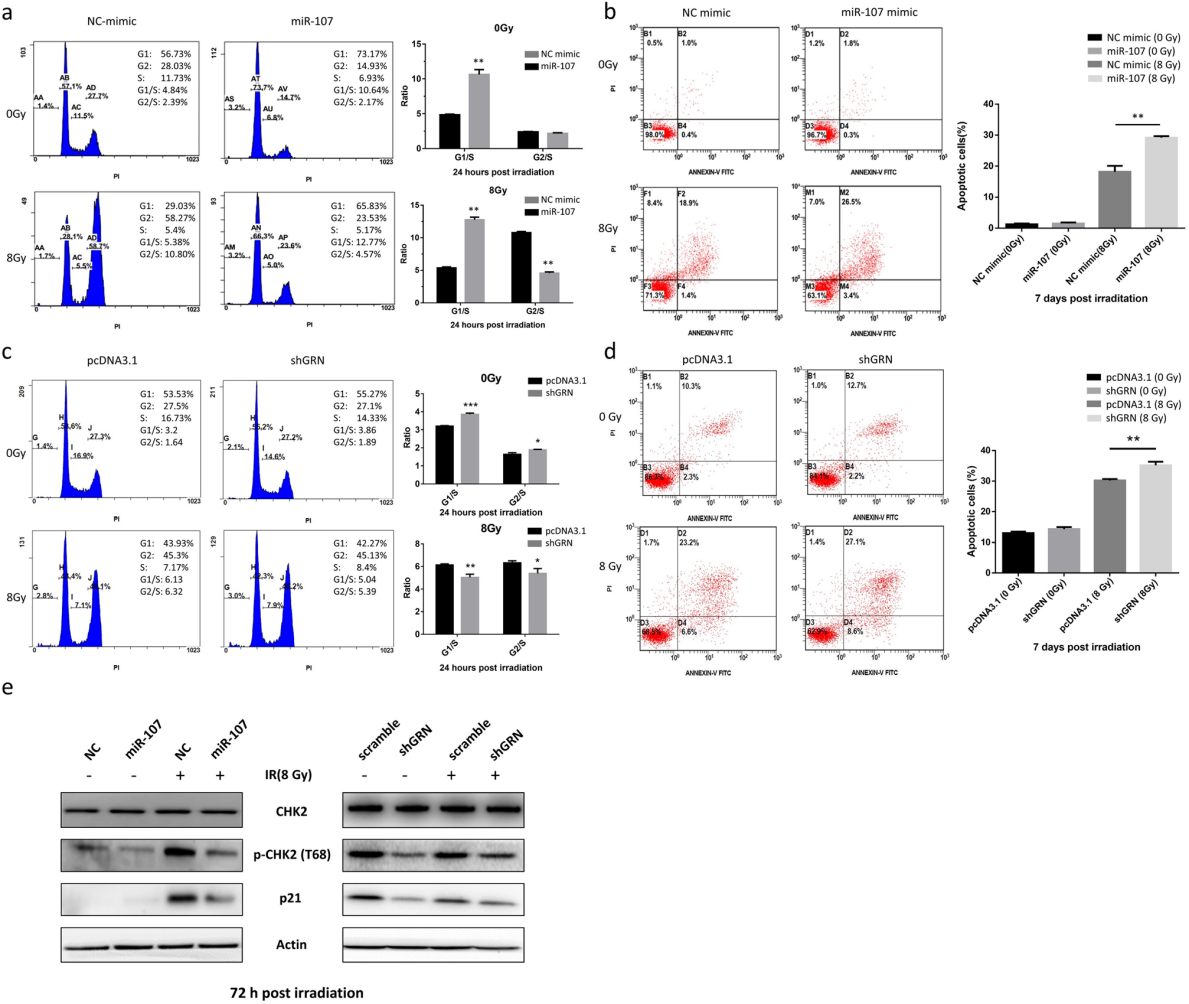
MiR-107 and shGRN altered radiation-induced cell cycle distribution and delayed apoptosis by repressing p21 expression and CHK2 phosphorylation in PC-3 cells. (**a**) Cell cycle distribution in PC-3 cells transfected with miR-107 mimic or negative control (NC), and each group was treated with 0 or 8 Gy IR. The bar graphs show the relative G1/S and G2/S ratios between the NC- and miR-107-transfected cells at 24 h. (**b**) Apoptosis was assayed by flow cytometry at 7 days after irradiation with 8 Gy. The graph indicates the percentage of apoptotic cells based on annexin-V-FITC and PI staining. (**c**) Cell cycle distribution in PC-3 cells transfected with shGRN or PCDNA3.1 (scramble), and each group was treated with 0 or 8 Gy IR. (**d**) Apoptosis was assayed at 7 days after irradiation with 8 Gy by annexin-V-FITC and PI staining. (**e**) Overexpression of miR-107 (left) or knockdown of *GRN* by shGRN (right) reduced p21 expression and CHK2 phosphorylation (Thr68) in response to IR at 72 h. Data were representative of more than three independent experiments, with each performed in triplicate. (*p < 0.05; **p < 0.01; ***p < 0.001).

The data above indicated overexpression of miR-107 enhanced the radiosensitivity of PC-3 cells by suppressing GRN. To determine whether the impact of miR-107 on cell proliferation and radiosensitivity is related to cell cycle control, we used flow cytometry to analyze the cell cycle distribution after IR in PC-3 cells transfected with NC mimic or miR-107 mimic. We found that miR-107 overexpression triggered cell cycle arrest in the G0/G1 phase in the absence of IR, which also decreased the proportion of cells in the S and G2/M phases at 24 h (Fig. 5a, upper row). After exposure to IR, miR-107 significantly increased the proportion of cells in the G0/G1 phase and decreased that in the G2/M phase. The increased G1/S ratio (> 2 fold) after receipt of 8 Gy indicated that miR-107 overexpression might enhance radiosensitivity via inducing the cell cycle arrest at the G1 checkpoint (Fig. 5a, lower row). However, the decreased proportion of cells in the G2/M phase and the decreased G2/S ratio (< 1/2 fold) indicated that miR-107 overexpression might result in G2/M transit.

Furthermore, to verify the role of GRN as a key effector in modulating radiosensitivity regulated by miR-107, we also investigated the cell cycle distribution after IR in PC-3 cells transfected with scramble or shGRN. The *GRN* knockdown also triggered cell cycle arrest in the G0/G1 phase in the absence of IR, which also decreased the proportion of cells in the S and G2/M phases at 24 h (Fig. 5c, upper row). After exposure to IR, in contrast to miR-107, shGRN significantly decreased the proportion of cells in the G0/G1 phase, but remained decreased in the G2/M phase (Fig. 5c, lower row). The consistently decreased proportion of cells in the G2/M phase, and the decreased G2/S ratio, indicated that *GRN* knockdown might enhance radiosensitivity through G2/M transit.

To further elucidate the mechanism of the suppressive effect of miR-107 and shGRN, annexin V binding assays were used to evaluate irradiation-induced apoptosis, which was calculated by subtracting the percentage of annexin V-positive cells with IR from that of the annexin V-negative group without IR. As shown in Fig. 5, miR-107 (Fig. 5b) and shGRN (Fig. 5d) consistently revealed a unique type of delayed-onset apoptosis at day 7 post-IR in PC-3 cells. These results suggest that, after IR, either miR-107 overexpression or *GRN* knockdown leads to altered cell cycle distribution and delayed apoptosis, which eventually enhances radiosensitivity.

**Figure 6:**
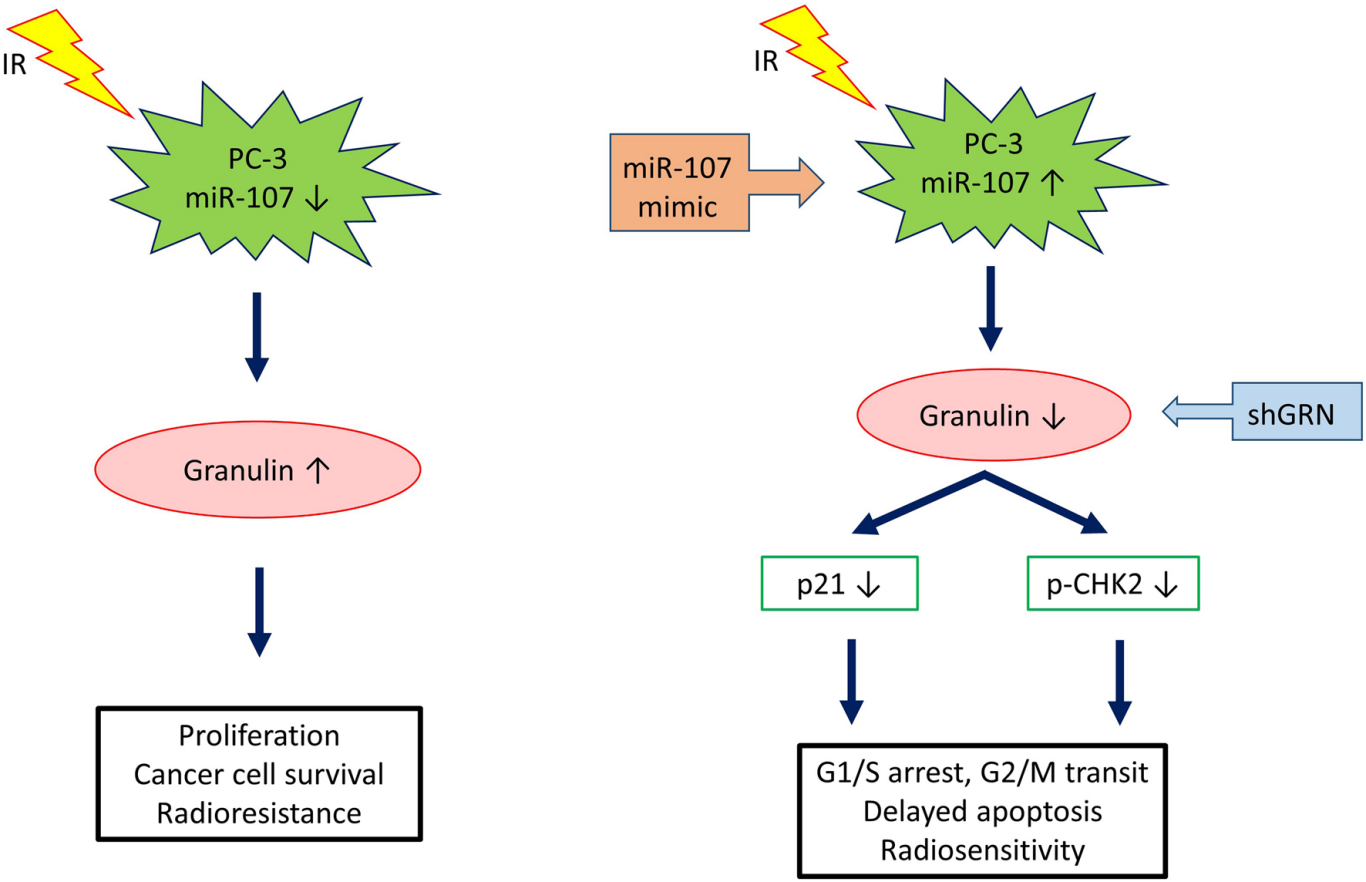
A schematic diagram illustrating miR-107 and GRN function in the balance between radiosensitivity and radioresistance in PC-3 cells. Left: MiR-107 is repressed and GRN induced in PC-3 cells following exposure to IR. p21 and CHK2, both cell cycle regulators, is activated or phosphorylated in response to DNA damage induced by IR, resulting in G1/S and G2/M arrest, which allows for repair of DNA damage and maintenance of genomic integrity. Right: Elevated miR-107 represses GRN expression by directly targeting GRN mRNA. Upon miR-107 overexpression and *GRN* knockdown, flow cytometry showed more prominent G1/S arrest and/or G2 check point abrogation (G2/M transit), and annexin V binding assays revealed delayed apoptosis after IR. Consequently, miR-107 sensitizes cancer cells to IR by down-regulating the p21 pathway or directly repressing CHK2 phosphorylation to modulate cell cycle checkpoint function.

Furthermore, to investigate the possible molecular mechanisms of miR-107/GRN-induced cell cycle arrest and delayed-onset apoptosis after IR exposure, several proteins associated with the DNA damage response (p-ATM, γ-H2AX), survival (p-Akt, p-ERK), apoptosis (PARP, bcl-2), and cell cycle regulation (cyclin D, p21, p-CHK2) were examined by western blotting. Most of these proteins showed no differences between cells with and without miR-107 overexpression after IR (supplemental Fig. 6), except p21 and phosphorylated CHK2 (p-CHK2). As shown in Fig. 5e, overexpression of miR-107 decreased the expression of p-CHK2 in the absence of IR, but did not change the level of p21 in the absence of IR. After exposure to IR, p-CHK2 and p21 were significantly increased in control cells (NC mimic), but decreased again in the presence of miR-107 mimic. Knockdown of *GRN* achieved similar results (Fig. 5e, right). These results suggest that miR-107 overexpression or *GRN* knockdown enhances the radiosensitivity of PC-3 cells through cell cycle regulation (including G1/S arrest and/or G2/M transit) by regulating p-CHK2 and p21, and leads to delayed apoptosis (Fig. 6).

## Discussion

Tumor relapse owing to radioresistance occurs in a certain proportion of prostate cancer (PCa) patients undergoing radiotherapy. Thus, therapeutic strategies to ameliorate the response to irradiation in PCa need to be developed. Radioresistance may originate from many biological processes including deregulated signaling pathways, mutations of oncogenes and/or tumor suppressors, disordered apoptosis and cell cycle checkpoints, and abnormal DNA damage response^32^. Mounting evidence has demonstrated a close link between reactive patterns of miRNA expression and radioresistance in various cancers^7–10,33,34^. Recent studies have shown miRNA can dominate tumor radiosensitivity by affecting signal transduction pathways, DNA damage repair, tumor microenvironment, apoptosis, and cell cycle checkpoints^35–37^. In this study, we found miR-107 expression was significantly downregulated upon exposure to ionizing radiation (IR) with a time-dependent pattern in PC-3 cells. Thus, we further characterized the role of miR-107 in regulating radiosensitivity in PC-3 cells. Additionally, we identified the miR-107 target granulin (GRN) as an important cell cycle regulator and activator of radiation-induced apoptosis.

Biological functions of the miR-15/107 superfamily have been widely studied including cellular metabolism, angiogenesis, stress, and cell division^11^. Thus, certain human diseases, such as neurodegenerative diseases, heart disease and neoplasia, are closely associated with dysregulation of this group of miRNAs. Results from several studies have confirmed the value of miR-107 in the therapeutic field of certain cancers. Molina-Pinelo and colleagues found that miR-107 and miR-99a-3p may be applied to estimate response to chemotherapy with standard fluoropyrimidine-based regimens in patients with metastatic colorectal cancer^38^. Teng and colleagues demonstrated that the expression of miR-107 could be suppressed by Lin28, a highly conserved RNA-binding protein, and resulting in up-regulation of C-myc and P-gp and down-regulation of cyclin D1, and subsequently leading to chemo-resistance in gastric cancer cells^39^. However, the investigations of miR-107 in radiotherapy are limited and its role in this context is elusive. Our study is the first to demonstrate miR-107 can act as a radiosensitizer in PCa cells. In our studies, overexpression of miR-107 suppressed PCa cell survival and enhanced growth inhibition after exposure to irradiation (Fig. 1d,e and supplemental Fig. 2a).

MiRNAs exert their functions through regulating the mRNA of their target genes. Of the target genes regulated by miR-107, *GRN* is notable due to its overexpression in several cancer cell lines, including lung, breast, hepatic, gastrointestinal, and genitourinary cancers. GRN was also reported to be overexpressed in prostate cancer tissues^26^, and later in several human cancers^40–42^, whereas normal tissues express little or no GRN. The highly expressed GRN drives tumor progression, since it stimulates cellular responses containing angiogenesis, invasion, proliferation, migration, malignant transformation, immune evasion and resistance to anticancer drugs^43^. Monami and colleagues suggested that GRN in PCa may play a role in facilitating progression to the stage of androgen-independent/hormone-refractory tumor, which is more aggressive and highly metastatic^27^. In this study, we had similar findings and found that GRN expression is more prominent in the androgen-independent prostate cancer cell lines PC-3 and DU145 than in the androgen-dependent prostate cancer cell line LNCaP (Supplementary Fig. 3). However, little is known about the role of GRN in radiation responses.

In the present study, we found GRN expression increased after exposure to IR in a time-dependent manner in PC-3 cells (Fig. 2). In addition, our data showed either miR-107 overexpression (Fig. 1) or knockdown of *GRN* (Fig. 3) could consistently achieve inhibition of growth and survival in PC3 cells after IR exposure, and overexpression of GRN attenuated the suppression effect of miR-107 mimic (Fig. 4). Taken together, these results show GRN plays a role in radioresistance and GRN suppression is a key player in radiosensitivity enhanced by miR-107 in PC-3 cells.

The exact mechanism by which GRN suppression enhanced the radiosensitivity of PC-3 cells is still unclear. GRN has multiple biological functions and stimulates several important signaling pathways, notably the PI3K/Akt and ERK pathways^27,44–46^, and activates the level of cell cycle proteins, such as cyclin D1 and cyclin B^47,48^. These signaling pathways are known to regulate radiosensitivity^3^; thus, GRN might induce radioresistance in PCa cells through the regulation of these pathways. However, in our study, the proteins associated with PI3K/Akt, ERK and cyclin D1 after IR were not significantly changed upon either miR-107 overexpression or *GRN* knockdown (supplemental Fig. 6), which means radiosensitivity might be achieved via different signal pathways or mechanisms in PC-3 cells.

To unveil the mechanism of radiosensitivity regulated by miR-107-GRN, we used flow cytometry to analyze cell cycle distribution at different time points (0, 12, 24, 48, and 72 h) after transfection of miR-107 mimic in PC-3 cells post IR, and found that prominent G1/S arrest and G2/M transit occurred early at 24 h (Fig. 5a). Notably, in PC-3 cells transfected with short hairpin GRN (shGRN), the G1/S arrest was decreased, but G2/M transit remained (Fig. 5c). However, the annexin V binding assays (Fig. 5b,d) revealed significant apoptosis consistently at day 7 after IR (i.e., delayed-onset apoptosis). Thus, the different G1/S ratio after receipt of 8 Gy implied that miR-107 overexpression might affect more cell cycle regulators (e.g., G1 checkpoint) than *GRN* knockdown. Nevertheless, these findings support that miR-107-GRN enhances radiosensitivity through affecting cell cycle and achieving delayed onset apoptosis in PC-3 cells after IR. Furthermore, western blotting analysis of cell cycle regulators showed suppressed protein levels of p21 and p-CHK2 in PC-3 cells transfected with either miR-107 mimic or shGRN after IR (Fig. 5e). p21 is a multifunctional protein, and is involved in regulating many cellular processes, including cell cycle control. The expression of p21 is regulated by both p53-dependent and p53-independent pathways^49^. Some studies showed p21 was required for maintaining the G2 checkpoint^50,51^. Chang and colleagues showed that p21 was essential for long-term G2 arrest, and confirmed that downregulation of p21 by siRNA lead to the arrested cells partly getting through the G2 block, and entering into the M phase with DNA damage in human uveal melanoma 92-1 cells^52^. In addition, the CHK2 kinase has a pivotal role in the checkpoint response to DNA damage that is associated with cell cycle arrest and apoptosis. Activated CHK2 propagates the DNA damage signal to several effector proteins involved in cell cycle arrest, apoptosis, and DNA repair^53–55^. Several lines of evidence, however, imply that combination of CHK2 inhibition with genotoxic agents (chemotherapeutics or IR) might have therapeutic value^56^. CHK2 inhibition has been demonstrated to attenuate cell cycle checkpoints (S- and G2-phases) induced by DNA damage and to enhance apoptotic activity in HEK-293 cells^57^. Inhibition of CHK2 can sensitize proliferating cells to doxorubicin-induced apoptosis and also abrogates the G2 checkpoint leading to mitotic catastrophe following DNA damage^58^. In another report, targeting CHK2 by using siRNA or a dominant-negative form of CHK2 kinase could prevent survivin release from the mitochondria and enhance apoptosis caused by doxorubicin^59^ or IR-induced DNA-damage. Taken together, these evidences support a linkage between suppressed p21/p-CHK2 levels and delayed apoptosis, most likely resulting from mitotic catastrophe, although this still needs more experiments to validate this. Mitotic catastrophe is considered the important mechanism of cell death in solid tumors respond to clinical radiotherapy. The cell death following mitotic catastrophe is characterized by a delay, occurring within two to six days following IR^60,61^.

To the best of our knowledge, this is the first report about the intersecting roles of miR-107 and granulin (GRN) in radiosensitivity of PC-3 cells. We report a novel role for miR-107 as a key regulator of radiation-induced apoptosis and show miR-107 sensitizes PC-3 to ionizing radiation by inhibiting GRN expression. These results may enrich our knowledge of connections between miRNA and tumorgenesis. Furthermore, we find both miR-107 and GRN responded to irradiation and either overexpression of miR-107 or knockdown of *GRN* sensitized PC-3 cells to irradiation. Collectively, these data suggest an unrecognized mechanism of miR-107/GRN regulation in response to ionizing radiation, which provide a potential therapeutic strategy for improving radiosensitivity of prostate cancer.

## Materials and methods

### Cell culture and irradiation treatment

The human prostate cancer cell lines, PC-3, LNCaP and D-U145 were purchased from the Bioresource Collection and Research Center (Hsinchu, Taiwan). PC-3 cells were maintained in F12K medium supplemented with 7% fetal bovine serum (FBS) and 1% penicillin and streptomycin mixture. LNCaP and DU-145 cells were maintained in RPMI-1640 medium supplemented with 10% FBS and 1% penicillin/streptomycin. Cells were grown in a 5% CO2 humidified atmosphere at 37 °C. Human Embryonic Kidney (HEK)-293 T cells were provided by Dr. Shau-Ping Lin (Institute of Biotechnology, National Taiwan University), and were maintained in DMEM medium (GIBCO) supplemented with 10% FBS and 1% penicillin/streptomycin.

Irradiations was performed by a ^137^Cs irradiator. The dose rate was 0.05017 Gy/sec. Cells in exponential growth were irradiated at room temperature, and non-irradiated culture cells were handled in parallel with the irradiated samples.

### Cell infection and transfection

PC-3 and DU-145 cells were plated in antibiotic medium at a density of 2 × 10^6^ cells in 10 cm dishes and to adhere overnight. Cells were transfected the next day with 10 nmol/L of mirVana™ miRNA Mimic (Ambion, Austin, TX) referent to has-miR-107 and negative control (NC) using Lipofectamine RNAiMAX (Invitrogen) according to manufacturer’s protocol. Cells were incubated for 48 h (h) for further experiment, and the medium was replaced with a fresh medium at 24 h.

PC-3 cells were transfected with miR-107 inhibitor (mimic) (Thermo Fisher scientific, USA) according to manufacturer’s protocol. After transfection, the mRNA and protein level of GRN were examined by qRT-PCR and western blotting.

To generate a construct expressing GRN, *GRN* cDNA was generated from PC-3 genomic DNA using qRT-PCR. The DNA fragments were subcloned into the expression vector pcDNA™3.1^(−)^ (Invitrogen, USA) using the EcoRI and NotI sites. The cDNA sequences were confirmed by DNA sequencing, and contained only ORF of *GRN* mRNA (absence of 3′UTR). Cells were transfected with plasmid pcDNA3.1-GRN, 3 μg, using TransIT ®-2020 Transfection Reagent (Mirus Bio LLC). Cells were then incubated for 48 h in an antibiotic medium for further experiments.

### Virus production and cell infection

HEK-293T cells (4 × 10^6^) were co-transfected with transfer vector plasmid (shGRN clones, 8 μg), packaging plasmid (psPAX2, 6 μg), and envelope plasmid (pMD2G, 2 μg), using TransIT®-2020 Transfection Reagent (Mirus Bio LLC). The shGRN clones (TRCN000115978 and TRCN000115979) and the scramble (ASN0000000001) clone were shRNAs specific for the human GRN gene, and were purchased from the National RNAi Core Facility at the Institute for Molecular Biology, Academia Sinica, Taiwan. After transfection for 72 h, the supernatants containing virus particles were collected and stored at − 80 °C. For cell infection, the virus particles and Polybrene were added to PC-3 cells (4 × 10^4^) in a 24 well plate, and then centrifuged at 2,300 revolutions per minute (rpm) for 60 min at room temperature.

### Clonogenic assay

Cells were plated at 60–80% confluency in 25 cm^2^ tissue culture flasks and allowed to adhere overnight followed by radiation exposure from 0 to 8 Gy as indicated. After irradiation, cells were trypsinized and plated in quadruplicate in 6 cm dishes at a density of 100–20,000 cells/dish. Cells were incubated for 14 days and then washed with PBS, fixed with 3:1 methanol-glacial acetic acid, and stained with 0.5% crystal violet. Colonies with ≧ 50 cells were counted. Plating efficiency (PE) and surviving fraction (SF) were calculated by following formula:

$$ \begin{aligned} & {\text{PE}} = {\text{number}}\;{\text{(no}}.{)}\;{\text{of}}\;{\text{colonies}}\;{\text{formed/no}}.\;{\text{of}}\;{\text{cells}}\;{\text{seeded }}*{1}00\% \\ & {\text{SF}} = {\text{no}}.\;{\text{ of }}\;{\text{colonies}}\;{\text{ formed }}\;{\text{after}}\;{\text{ irradiation }}/{\text{no}}. \, \;{\text{of}}\;{\text{ cells }}\;{\text{seeded }}*{\text{ PE}}. \\ \end{aligned} $$

The clonogenic surviving curve for each data set was fitted to a linear-quadratic model using GraphPad Prism 6.0 software (La Jolla, CA, USA).

### Western blotting

Cells were washed twice with PBS and resuspended with RIPA lysis buffer (Millipore) containing protease inhibitors. Protein concentration was determined by the Pierce™ BCA Protein Assay kit. Protein samples (40 μg) were loaded on a 10% SDS gel and transferred to a methanol-activated PVDF membrane. The membrane was blocked with 5% nonfat milk in Tris-buffered saline overnight at 4˚C. Then, the membrane was incubated for 2 h at room temperature with primary antibodies against Action (β-actin, Thermal Fisher, USA), total CHK2 (Cell Signaling, USA), phospho-CHK2 Thr68 (Cell Signaling, USA), p21 (Cell Signaling, USA) and GRN (Invitrogen, USA) for 2 h at room temperature. After washing with PBS three times and incubation with a secondary antibody for 1 h, blotted proteins were visualized by enhanced chemiluminescence (Millipore) with the BioSpectrum Imaging System (UVP, Upland, CA, USA).

### RNA extraction, reverse transcription, and qRT-PCR quantification

Total RNA was extracted from PCa cell lines by TRIZOL reagent (Invitrogen). RNA concentration was measured by an ND-1000 NanoDrop spectrophotometer (Thermo Fisher, USA). For the Universal ProbeLibrary probe assay, miRNA (1 μg) was reverse transcribed into cDNA with 50 nM stem loop RT primer by SuperScript™ II reverse transcriptase (Invitrogen, USA) for *miR-107* mRNA detection, and the RT stem loop primers used for *miR-107* was 5′ GTTGGCTCTGGTGCAGGGTCCGAGGTATTCGCACCAGAGCCAACTGATAG 3′. qRT-PCR was performed by a LightCycler® 480 instrument (Roche, Germany) with a miR-107 specific primer (Forward: 5′ GCAAGCAGCATTGTACAGGG 3′), Universal Reverse primer (Forward: 5′GTGCAGGGTCCGAGGT3′), Universal Probe Library Probe #21 (Roche, Germany), and KAPA PROBE FAST qPCR master mix (Kapa Biosystems, Boston, USA). For the SYBR Green assay, total RNA (1 μg) was transcribed into cDNA with random primers (Applied Biosystems) by a High Capacity cDNA reverse transcriptase kit (Applied Biosystems) for *GRN* and *PANK1* mRNA detection. The mRNA expression levels were detected by a 7,300 Fast Real-Time PCR instrument (Applied Biosystems) with *GRN* primers (Forward: 5′ CAGGGGTACCAAGTGTTTGC 3′; Reverse: 5′ AATTTGGTTAGGGGGAGGTG 3′) in Power SYBR® Green Master Mix (Applied Biosystems). The miRNA and mRNA expression levels were normalized to RNU44 and 18srRNA, respectively, using the 2^-ΔΔCt^ method.

### Cell proliferation assay

Cells were seeded at a density of 4 × 10^4^ cells/mL in a 24-well plate with six replicates for each group and allowed to grow for 24, 48, 72, and 96 h. At the indicated time point, culture medium was removed and replaced with thiazolyl blue tetrazolium blue (MTT) for 4 h at 37 °C. At the end of incubation, the supernatants were removed and replaced with 400 μL of DMSO (Sigma, USA) for each well. The absorbance value was measured at 570 nm by a VICTOR multi label plate reader (PerkinElmer, MA, USA).

### Flow cytometry analysis of the cell cycle

Cells were plated at 40–50% confluency in 75 cm^2^ tissue culture flasks and allowed to adhere overnight followed by radiation exposure from 0 to 8 Gy. After irradiation at the indicated doses, cells were grown for 0–96 h and harvested. Samples were trypsinized, resuspended in PBS, and fixed with cold 75% ethanol overnight at 4 °C. The fixed samples were centrifuged at 2,200 rpm for 5 min, then washed twice with cold PBS and resuspended in 500 μL of PI staining solution (BD Biosciences) for 30 min. The DNA content of labeled cells was measured by Cytomic FC 500 flow cytometer (Beckman Coulter, Inc).

### Annexin V analysis for apoptosis

We performed annexin V analysis for apoptosis according to the protocol as previously reported^30^. The FITC Annexin V Apoptosis Detection Kit (BD Pharmingen, San Jose, CA, USA) was used to detect apoptotic cells by flow cytometry. Cells were exposed to 8 Gy of IR, and were harvested at different time points for 7 days after exposure. Annexin V binding buffer was used to resuspend cells, and the cell suspensions were stained with FITC-annexin V and PI staining solution for 15 min at room temperature. The apoptotic/necrotic cells were analyzed with a FACS Calibur flow cytometer (Becton Dickinson). The IR-induced apoptosis was calculated by subtracting the percentage of annexin V-positive cells with IR from that of the corresponding group without IR.

### Statistical analysis

Statistical analysis was performed using GraphPad Prism 6.0 software (La Jolla, CA, USA). A two-tailed Student’s t-test was applied to all the data in this study. Values were considered to be significant if *p* < 0.05. All values in the figures are presented as the mean ± SEM. Data were representative of more than three independent experiments, with each performed in triplicate.

## Supplementary information

Supplementary information.
