# Supplementary material for: MicroRNA-107 enhances radiosensitivity by suppressing granulin in PC-3 prostate cancer cells
Source: Sci Rep. 2020 Sep 3;10:14584. doi: 10.1038/s41598-020-71128-1 (PMC7471693; doi:10.1038/s41598-020-71128-1)
Supplement: Supplementary file 1 — Supplementary information. [file 41598_2020_71128_MOESM1_ESM.pdf]

MicroRNA-107 Enhances Radiosensitivity by Suppressing Granulin in PC-3 Prostate Cancer Cells

Hua-Cheng Lo[1,2], Jia-Hao Hsu[1,2], Liang-Chuan Lai[3,4], Mong-Hsun Tsai[4,5], Eric Y. Chuang[2,4,6]

[1]Division of Urology, Tri-Service General Hospital Song-Shan Branch, National Defense Medical Center, [2]Graduate Institute of Biomedical Electronics and Bioinformatics, [3]Graduate Institute of Physiology, College of Medicine, [4]Bioinformatics and Biostatistics Core, Center of Genomic and Precision Medicine, [5]Institute of Biotechnology, National Taiwan University; and [6]Biomedical Technology and Device Research Laboratories, Industrial Technology Research Institute, Hsinchu, Taiwan

Corresponding authors: Eric Y. Chuang, Sc.D., EMBA

Professor

Graduate Institute of Biomedical Electronics and Bioinformatics

Department of Electrical Engineering

National Taiwan University,

Taipei 106, Taiwan.

Phone: 886-2-3366-3660; Fax: 886-2-3366-3682.

E-mail: chuangey@ntu.edu.tw

Supplementary Information

Supplementary Figures and Legends

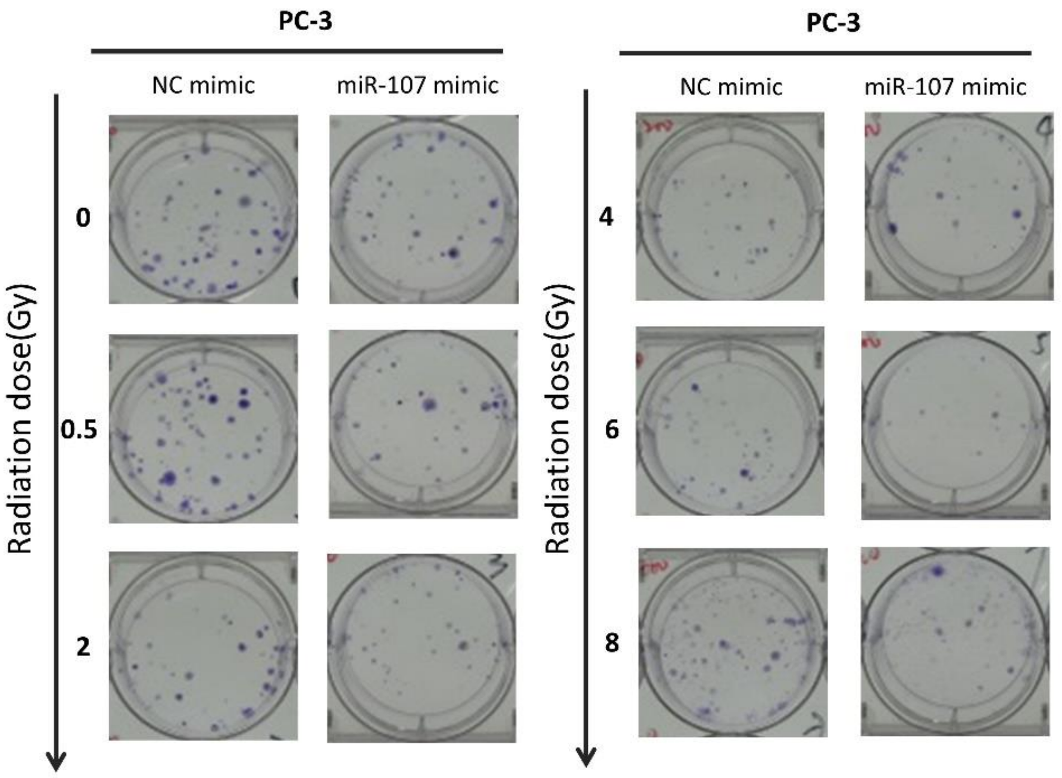

Supplementary Figure 1. MiR-107 sensitized PC-3 cells to IR exposure. Photographs of colonies formed by PC3 cells after IR (0, 0.5, 2, 4, 6, and 8 Gy) in 6-well plates. NC mimic plates showed 61.7, 57.7, 46, 47.3, 62.3, and 20 clones formed after seeding 100, 100, 100, 200, 800, and 2,500 cells, respectively. MiR-107 mimic plates revealed 48, 46.7, 51.7, 40.3, 27, and 14 formed after seeding 100, 100, 200, 400, 1,400, and 10,000 cells, respectively. Data were representative of more than three independent experiments, with each performed in triplicate.

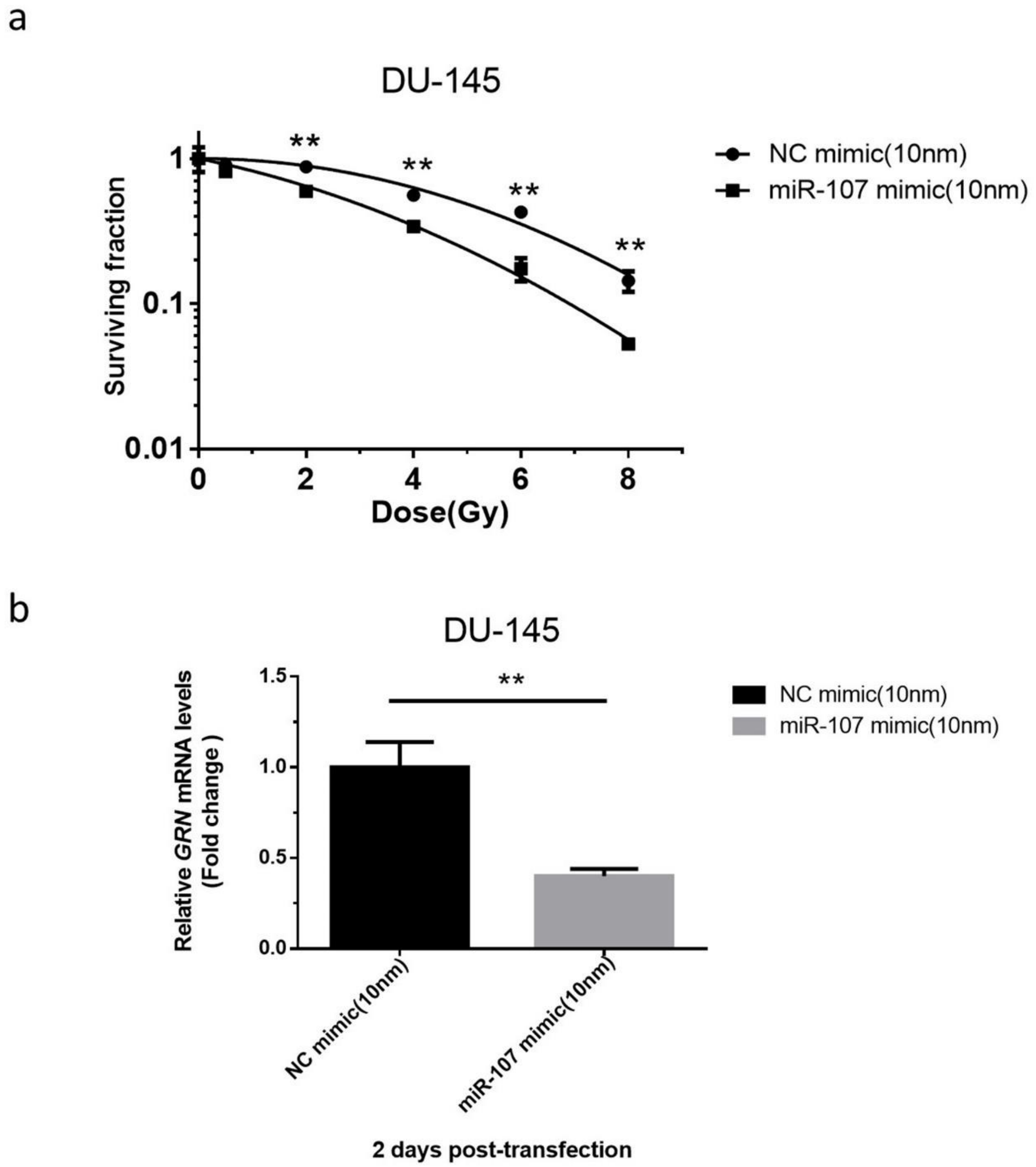

Supplementary Figure 2. Overexpression of miR-107 enhanced radiosensitivity of DU-145 cells and down-regulated GRN. (a) Colony formation of DU-145 cells transfected with miR-107 or NC after IR. (b) Relative expression of GRN mRNA after miR-107 treatment for 48 h. Data were representative of more than three independent experiments, with each performed in triplicate. (**$p < 0.01$)

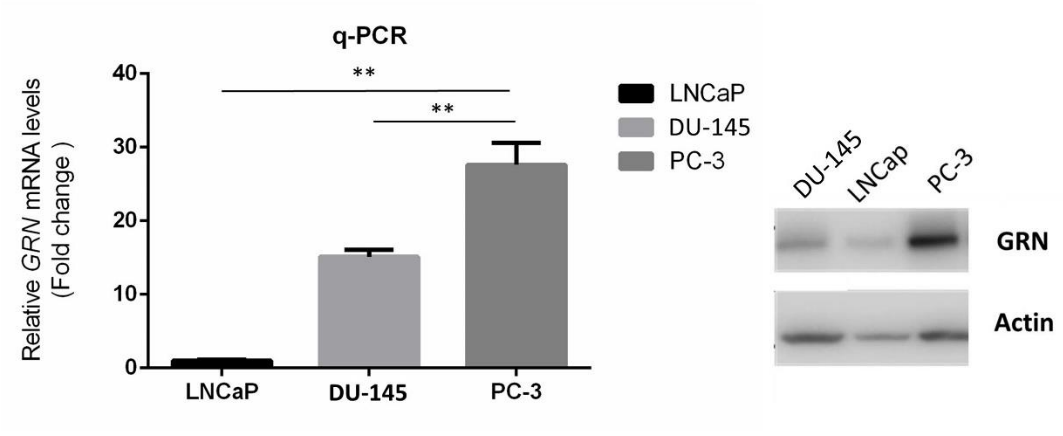

Supplementary Figure 3. GRN expression is cell line-specific. Relative mRNA (left) and protein (right) expression levels of GRN in LNCaP, DU-145, and PC-3 PCa cells were detected by qRT-PCR and western blotting. Data were representative of more than three independent experiments, with each performed in triplicate. (** $p < 0.01$)

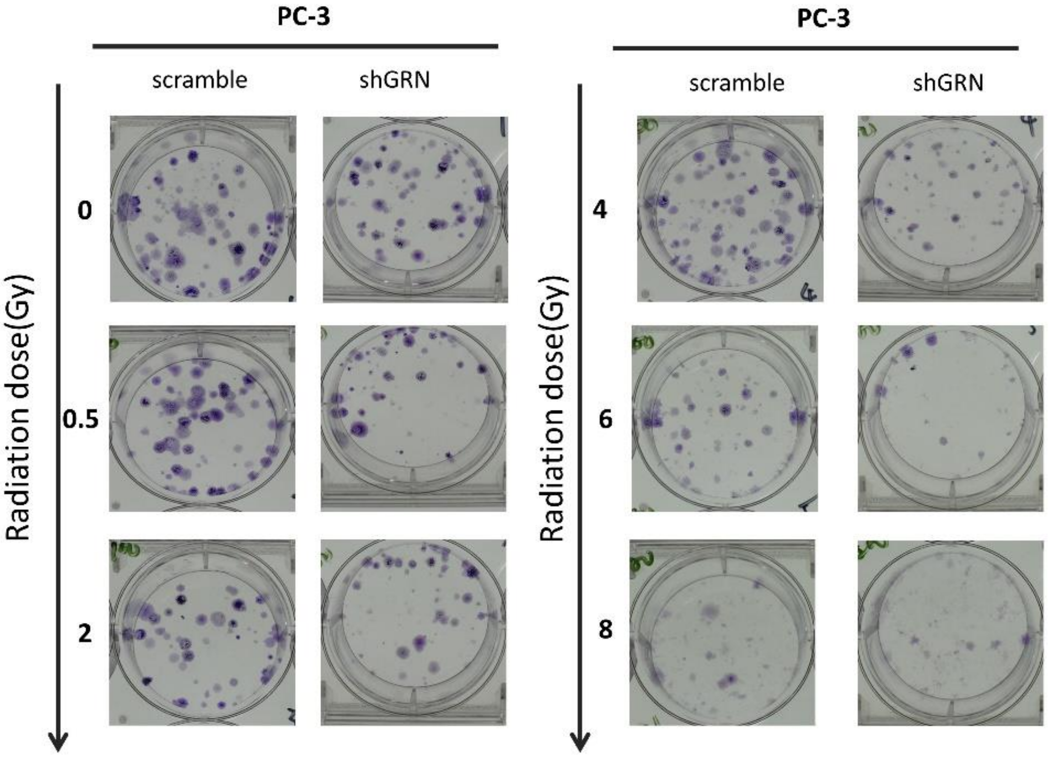

Supplementary Figure 4. GRN knockdown sensitized PC-3 cells to IR exposure. Photographs of colonies formed by PC-3 cells after IR (0, 0.5, 2, 4, 6, and 8 Gy) in 6-well plates. Scramble plates showed 65.7, 75, 52, 88, 71.7, and 74 clones formed after seeding 100, 100, 100, 300, 600, and 3000, respectively. shGRN plates revealed 66, 59, 56.7, 63, 36, and 37, respectively, formed after seeding 200, 200, 300, 600, 1,500, and 15,000 cells, respectively. Data were representative of more than three independent experiments, with each performed in triplicate.

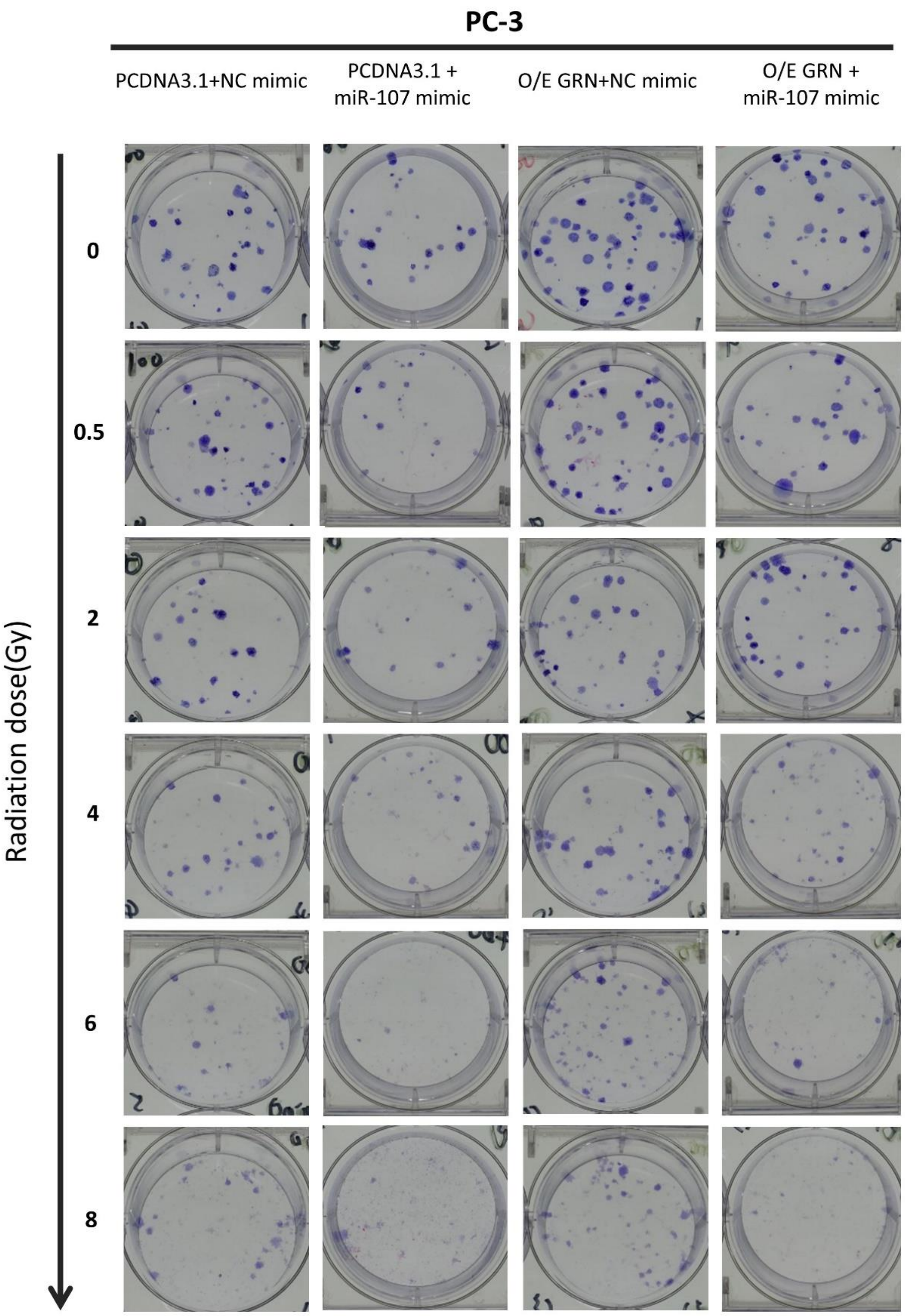
PC-3
PCDNA3.1+NC mimic
PCDNA3.1 + miR-107 mimic
O/E GRN+NC mimic
O/E GRN + miR-107 mimic
Radiation dose(Gy)
0
0.5
2
4
6
8

Supplementary Figure 5. GRN overexpression rescued survival repressed by miR-107 in PC-3 cells after IR exposure. Photographs of colonies formed by PC-3 cells after irradiation (0, 0.5, 2, 4, 6, and 8G y) in 6-well plates. pcDNA3.1+NC mimic plates showed 43.3, 41.3, 27.3, 31.7, 30.7, and 39.7 clones formed after seeding 100, 100, 100, 200, 800, and 2,000 cells, respectively. pcDNA3.1+miR-107 mimic plates showed 27, 22.7, 32.7, 29.3, 19, and 17 clones formed after seeding 100, 100, 200, 400, 1,400, and 10,000 cells, respectively. Overexpression (O/E) GRN+NC mimic plates revealed 53, 45.7, 41.7, 55, 68.3, and 57 clones formed after seeding 100, 100, 100, 200, 500, and 1,000 cells, respectively. O/E GRN+miR-107 mimic plates revealed 40.3, 33, 30.3, 41, 37, and 23.3 clones formed after seeding 100, 100, 100, 200, 800, and 2,000 cells, respectively. Data were representative of more than three independent experiments, with each performed in triplicate.

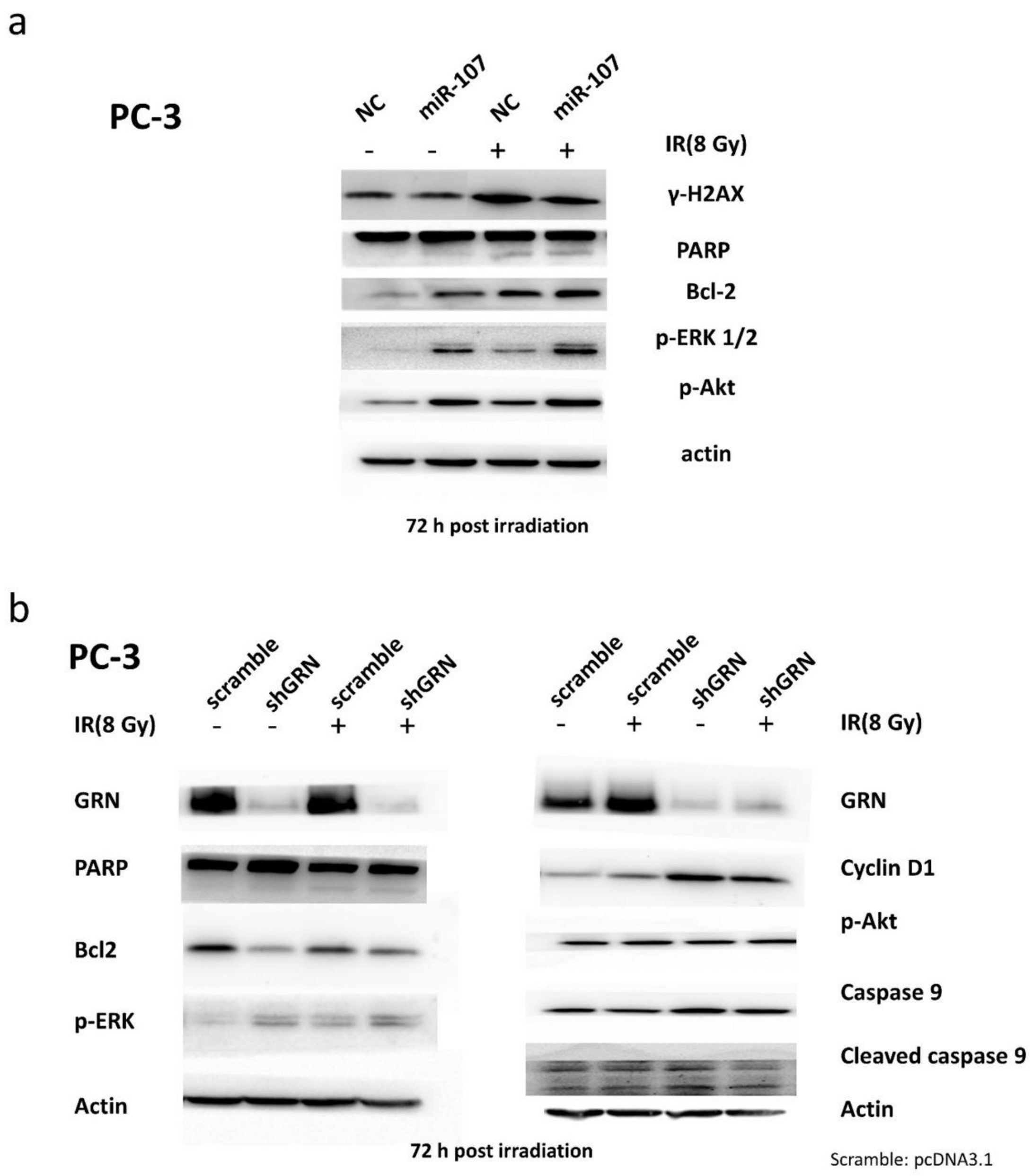

Supplementary Figure 6. Western blotting of several major proteins associated with radiation responses. The proteins associated with PI3K/Akt, ERK and cyclin D1 showed no significant difference upon either miR-107 overexpression (a) or *GRN* knockdown (b) after exposure to IR at 72 h. Data were representative of more than three independent experiments, with each performed in triplicate.
